# Forensic nursing in the emergency department: the distance between nurses’ performed role behaviors and their perception of behaviors’ importance

**DOI:** 10.1186/s12912-023-01682-2

**Published:** 2024-01-06

**Authors:** Somayeh Zare Emami, Virginia A. Lynch, Marjan Banazadeh

**Affiliations:** 1 Student of Emergency Nursing, School of Nursing, Research Committee Student, School of Nursing, Alborz University of Medical Sciences, Karaj, Iran; 2 University of Colorado, 514 Hopi Circle, Colorado SpringsDivide, CO 80814 USA; 3 Department of Nursing, School of Nursing, Alborz University of Medical Sciences, Taleghani Boulevard, Taleghani Square, Karaj, Iran

**Keywords:** Emergency department, Frequency, Forensic nursing, Importance, Nurses, Role

## Abstract

**Background:**

Emergency department nurses often deal with victims of violence and trauma. In the emergency department, the main focus is on saving lives and stabilizing patients' conditions. The next important task is to preserve any valuable evidence that could potentially help identify a crime. It is important to describe how nurses currently practice in the emergency department and perceive their role in caring for forensic patients. This study aimed to investigate the frequency of performance and perception of the importance of forensic nursing role behaviors among emergency department nurses as well as the correlation between mean scores of performance and perception.

**Methods:**

This is a cross-sectional correlational descriptive study. This non-observational survey study used a questionnaire to investigate the frequency of performance and perception of the importance of forensic nursing role behaviors among 274 emergency department nurses.

**Results:**

The total mean scores for frequency of performed behaviors and their perceived importance were 2.36±0.65 and 4.23±0.64 respectively. The overall mean scores of importance were significantly higher than frequency. There was a significant correlation between the frequency of performance and perception of the importance of twenty-eight items (twenty-four positive correlations and 4 negative correlations) (*p<*0.05). The frequency of performed behaviors positively correlated with participants’ type of shift worked and their personal experiences of legal and judicial claims. The female gender of respondents positively correlated with behaviors’ perceived importance.

**Conclusion:**

The study revealed a significant discrepancy between the frequency of forensic nursing role behaviors performed and their perceived importance in the emergency department. This gap emphasizes the pressing requirement for forensic nursing subjects to be incorporated into graduate and undergraduate nursing curricula, as well as ongoing training programs and courses. It is crucial to establish and implement forensic nursing protocols for the care of trauma victims, and to foster collaboration between healthcare systems, law enforcement, and forensic investigators to streamline the process.

## Introduction

Patients with traumatic injuries visit emergency departments (ED) [1, 2]. They may suffer unintentional trauma caused by crashes like falls and motor vehicle accidents as well as intentional trauma caused by sexual assault, child or elder abuse, domestic violence, and suicide [3, 4]. Injuries and violence are significant causes of mortality and morbidity worldwide. Annually out of the 4.4 million deaths caused by injuries, 3.16 million result from unintended injuries, and 1.25 million are associated-violence injuries.

In Iran, road traffic crashes and falls are the most prevalent causes of injuries [5]. Violence is one of the five social harms [6] and associated injuries are the second most significant cause of mortality. While firearm use in violent situations has declined in Iran, the rate of deaths resulting from interpersonal violence among adults has increased between 1990 to 2015 [7]. About two-thirds of married women (67.3%) have experienced at least one type of domestic violence during their lifetime [8]. Soleimani et al 2021, estimated the prevalence of domestic violence during pregnancy in Iran to be 52% [9]. With the increasing elderly population, attention to issues related to elder abuse becomes important. The overall prevalence of elder abuse in Iran is 56.4%, and the most common types of elder abuse in order are emotional abuse (30.7%), psychological abuse (25.4%), neglect (25.1%), financial abuse (19.7%), physical abuse (13.1%), and abandonment (11.7%) [10]. Furthermore, the overall prevalence of physical abuse, emotional abuse, and neglect among Iranian children is reported to be 43.59%, 64.53%, and 40.94%, respectively [11].

Emergency department nurses are on the frontline to meet patients [12, 13]. Their role in triage and initial care emphasizes life-saving and patient condition stabilization [14]. They also provide secondary services to victims of trauma and violence [12]. Forensic nursing (FN) is a relatively new expertise that provides medico-legal care to patients injured by trauma and violence [15] by integrating nursing care with forensic science and offense inquiry [16]. Forensic nurses conduct medical-forensic examinations, take medical-forensic photographs, identify, collect, and preserve the chain of evidence, and then transfer it to law enforcement authorities for investigation and legal judgment of a crime [1, 17]. They also provide expert witness statements in court [18] and assist victims of abuse in reporting crimes [1, 17], and in cases of abuse, they coordinate with multidisciplinary teams to assess the situation [15, 19].

Working as a forensic nurse requires special skills and knowledge to recognize the signs and symptoms of violence and abuse [15]. Inadequate awareness of ED nurses may lead to misidentification of evidence and injustice [20, 21]. To effectively fulfill their role, ED nurses must possess more than just the ability to identify injury or evidence. They must also possess the necessary skills to provide high-quality, evidence-based care and meet the standard of care for their patients [22, 23].

Most ED nurses may believe that their primary role is a priority. They may be inadequately aware of their multiple roles which may create conflict and confusion about the FN role [24, 25]. Without a clearly defined role and set of skills recognition of the need to fill the role safely and at the standard of care is difficult. Clarification and recognition of the FN role are essential to define the scope of practice within and outside healthcare settings. It is important to describe how nurses currently practice in EDs and perceive their role in caring for forensic patients.

Lynch 1990 and Abdul and Brysiewicz 2009 identified ED nurses’ perceived FN role performance and importance. They found a low frequent implementation of FN behaviors and a gap between the frequency of performed behaviors and perception of behaviors’ importance [26, 27]. In Iran, Feizi Nazarloo et al (2017), in a study examining ED nurses’ knowledge of FN found ED nurses lacked enough knowledge of FN [28].

Despite the significant number of victims of violence and injuries in Iran [6] who require medico-legal care, the FN specialty is not recognized [28]. To our knowledge, there are no studies in Iran that have investigated ED nurses’ performance of FN role behaviors and their perceived behaviors’ importance. Therefore, we aimed to investigate the frequency of performance (FOP) and perception of the importance (POI) of forensic nursing role behaviors among emergency department nurses as well as the correlation between mean scores of performance and perception.

## Methods

### Design

This is a cross-sectional correlational descriptive study. In this non-observational survey study ED nurses evaluated their FOP and POI of FN role behaviors.

The sample consisted of ED nurses, recruited from 11 hospitals in Albroz province. These hospitals were government-run healthcare facilities and educational centers, with six EDs being general and the others specialized in cardiology, pediatrics, trauma, gynecology, and toxicology.

Using convenience sampling ED nurses who had worked in the ED for 6 months [27] and were interested in participating in the study were included.

### Instrument

Two questionnaires were used to collect data. First, a 12-item demographic questionnaire that was assumed to affect ED nurses’ performance of FN role behaviors and their perceived importance was designed. The items consisted of 3 categories as follows: (1) personal characteristics like gender, age, marital status, and level of education; (2) professional characteristics like total nursing experience, nursing experience in ED, type of ED, type of shift, and weekly working hours; (3) previous personal experiences related to formal FN education and personal experiences of legal and judicial claims. To examine the FOP and POI of FN role behaviors among ED nurses “the emergency department forensic nursing survey (EDFNS)” was used. This questionnaire was developed by Lynch in 1990. It consists of 39 items, of which 32 items examine the FOP and POI of FN role behaviors, 6 items examine the FOP and POI of forensic nurse specialists’ role behaviors, and 1 item examines the existence of physicians’ knowledge of forensic protocols. The FOP and POI of FN role behaviors were rated separately on a five-point Likert–type scale, ranging from (1=never, 2=seldom, 3=often, 4=almost always, and 5=always) as well as (1=very unimportant, 2=somewhat unimportant, 3=no opinion, 3=somewhat important, 5=very important) [26].

### Validity and reliability

Permission was obtained to translate and use the EDFNS from Dr. Lynch. The scale was translated from English to Persian using the forward-backward technique, following World Health Organization guidelines [29]. Two professional translators performed the initial translation and a primary Persian version was created after comparing their translations. This version was then back-translated to English by an expert translator who was unaware of the EDFNS. After careful cultural adaptation, the final version of the scale was developed [30]. Previous studies have confirmed the validity and reliability of the EDFNS [26, 27]. We assessed these characteristics in the Iranian context in this study, as no previous studies had done so. Content validity was assessed by 10 experts who evaluated the scale’s cultural and religious perspectives, grammar, wording, and item allocation. They independently rated each item for relevance, clarity, and simplicity. Based on their feedback, the scale was revised and POIlot-tested on 30 ED nurses. The test-retest Cronbach's α coefficient was 0.902 and the content validity index (CVI) was 0.92, indicating acceptable reliability and validity of the translated scale.

### Data collection and analysis

Questionnaires were given to ED nurses and they were informed about the study verbally and in writing. In total 350 sets of questionnaires were distributed between May and August 2022. Data were analyzed using SPSS 27, and a Kolmogorov-Smirnov test showed normal distribution. Descriptive statistics, Paired T-tests, ANOVA** (**Analysis of variance), and Spearman’s correlation tests were used. Variables were measured with mean scores on a scale of 1-5 (average=3). Items were ranked from highest to lowest. Statistical significance was set at *p<*0.05.

## Results

Out of 350 sets of questionnaires distributed, 274 were included in the study with a dropout of 76 (response rate, 78%) due to complete non-participation. The sample was aged between 21-51 years. About half (50.4%) were between 21-30 years old. They were mainly female (70.1%). Detailed demographic information is presented in Table 1.

**Table 1:** Participants’ demographic information (*N*=274)

**Variables**	**N (%)**
**Gender**	
Female	192 (70.1)
Male	82 (29.9)
**Marital status**	
Single	114 (41.6)
Married	159 (58.0)
Others (widows and divorced)	1 (0.4)
**Age (year)**	
21-30	138 (50.4)
31-40	122 (44.5)
41-50	13 (4.7)
More than 50 years	1 (0.4)
**Level of education**	
Bachelor	263 (96.0)
Master	10 (3.6)
Ph.D.	1 (0.4)
**Total nursing experience (year)**	
Less than 5	90 (32.8)
10-May	110 (40.1)
15-Oct	58 (21.2)
15-20	12 (4.4)
20-25	3 (1.1)
More than 25	1 (0.4)
**Nursing experience in ED (month/year)**	
6-12 m	63 (23.0)
1-10 y	179 (65.3)
10-20 y	32 (11.7)
More than 20 y	0 (0)
**Current position**	
ED supervisor	4 (1.5)
ED chief nurse	5 (1.8)
ED nurse	236 (86.1)
Triage nurse	29 (10.6)
**Type of shift**	
In Rotation	249 (90.9)
Morning	14 (5.1)
Evening	4 (1.5)
Night	7 (2.6)
**Weekly working hours**	
Less than 30	3 (1.1)
30-40	116 (42.3)
More than 40	155 (56.6)
**Type of ED**	
General	227 (82.8)
Poisoning	17 (6.2)
Trauma	18 (6.6)
Cardiology	12 (4.4)
Psychiatry	1 (0.4)
**Formal forensic nursing education**	
Yes	18 (6.6)
No	256 (93.4)
**Personal experiences of legal and judicial claims**	
Yes	16 (5.8)
No	258 (94.2)

### Descriptive results

The results of the experts’ ratings of EDFNS items regarding relevance, simplicity, and clarity indicated that the questionnaire achieved an acceptable content validity index (0.92).

### The FOP and POI of FN role behaviors

**Fig. 1:**
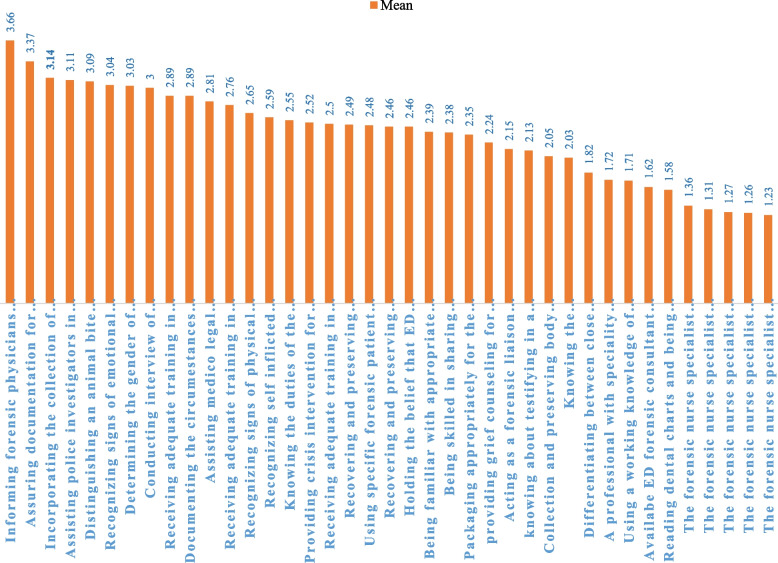
Ranking of the FOP of forensic nursing role behaviors

**Fig. 2:**
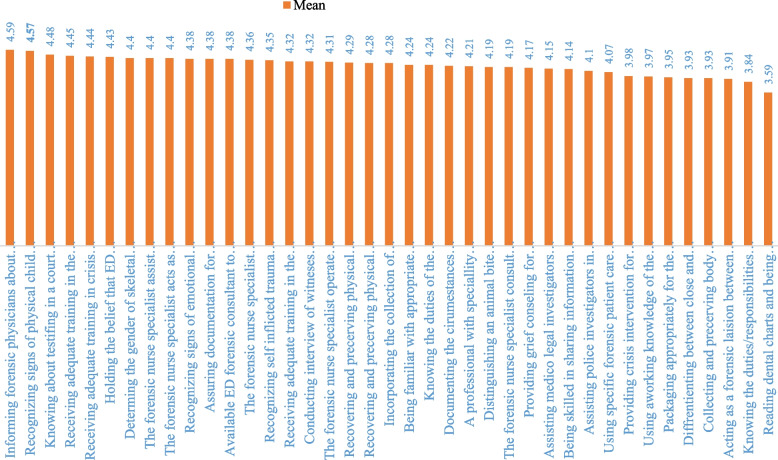
Ranking of the POI of forensic nursing role behaviors

The mean scores of frequency of performed (FOP) forensic nursing role behaviors ranged from 1.27 to 3.66 with a total mean score of 2.36±0.65. Thirty-one items gained a mean score below the average and only eight items scored above the average. The mean scores of perception of importance (POI) of forensic nursing role behaviors ranged from 3.59 to 4.59 with a total mean score of 4.23±0.64 and all items gained a mean score above the average. The average score for both variables was considered 3. The mean scores for importance were significantly higher than those for frequency, both for individual items and overall (Table 2, Figs. 1, 2).

**Table 2:** The frequency of FOP and POI of forensic nursing role behaviors and the correlation between their means

**Forensic nursing role behaviors**		**Frequency**			**Importance**			
		**Mean**	**SD**	**Rank**	**Mean**	**SD**	**Rank**	**Correlations/r**
1	Informing forensic physicians about admitting a patient who died on arrival without receiving care	3.66	1.56	1	4.59	0.87	1	0. 53^a^
2	Distinguishing an animal bite wound from a human one	3.09	1.34	5	4.19	0.96	25	0. 41^a^
3	Recovering and preserving physical trace evidence of a sexual assault	2.49	1.40	18	4.29	1.04	18	0. 30^a^
4	Recovering and preserving physical trace evidence of a sexual suspect	2.46	1.34	20	4.28	1.04	19	0. 26^a^
5	Determining the gender of skeletal remains brought to the ED	3.03	1.62	7	4.40	0.98	7	0. 31^a^
6	Knowing about testifying in a court of law in forensic cases	2.13	1.13	27	4.48	0.94	3	0. 11
7	Differentiating between close and distant gunshot wounds	1.82	1.05	30	3.93	1.18	35	0. 20^a^
8	Packaging appropriately for the preservation of physical evidence	2.35	1.26	24	3.95	1.06	34	0. 23^a^
9	Being familiar with appropriate documentation of death or injury	2.39	1.37	22	4.24	1.06	21	0. 23^a^
10	Recognizing signs of physical child abuse	2.65	1.14	13	4.57	0.76	2	0. 11
11	Recognizing signs of emotional battering/abuse	3.04	1.15	6	4.38	0.86	10	0. 25^a^
12	Reading dental charts and being aware of the forensic dental comparison	1.58	0.90	34	3.59	1.26	39	0. 28^a^
13	Knowing the duties/responsibilities of the police investigator	2.03	1.03	29	3.84	1.14	38	0. 22^a^
14	Knowing the duties of the coroner/medical examiner	2.55	1.23	15	4.24	0.95	22	0. 16^a^
15	Recognizing self-inflicted trauma versus inflicted by others	2.59	1.15	14	4.35	0.93	14	0. 17^a^
16	Being skilled in sharing information about sexual assault with law officers/medico-legal investigators	2.38	1.19	23	4.14	0.96	29	0. 24^a^
17	Assisting police investigators in their mission	3.11	1.27	4	4.10	1.05	30	-0. 45^a^
18	Assisting medico-legal investigators in their mission	2.81	1.30	11	4.15	1.01	28	0. 34^a^
19	Incorporating the collection of forensic evidence with patient care	3.14	1.36	3	4.28	0.93	20	0. 38^a^
20	Assuring documentation for investigation and legal protection of the nurse/patient	3.37	1.37	2	4.38	0.92	11	0. 34^a^
21	Using specific forensic patient care guidelines	2.48	1.29	19	4.07	1.00	31	0. 24^a^
22	Receiving adequate training in the notification of death	2.76	1.34	12	4.45	0.83	4	0. 15
23	Receiving adequate training in grief counseling	2.50	1.33	17	4.32	0.89	15	0. 12
24	Providing grief counseling for victims/their relatives.	2.24	1.24	25	4.17	0.99	27	0. 11
25	Receiving adequate training in crisis intervention.	2.89	1.30	9	4.44	0.82	5	0. 19^a^
26	Documenting the circumstances around the victim and interactions between patient/family/witnesses.	2.89	1.21	10	4.22	1.00	23	0. 32^a^
27	Acting as a forensic liaison between the hospital/victims, forensic investigators/law enforcement	2.15	1.28	26	3.91	1.18	37	0. 32^a^
28	Collecting and preserving body tissues and fluids as evidence	2.05	1.23	28	3.93	1.19	36	0. 26^a^
29	Providing crisis intervention for victims and their relatives	2.52	1.19	16	3.98	1.05	32	0. 22^a^
30	Conducting interviews of witnesses in suspected cases	3.00	1.31	8	4.32	0.91	16	0. 28^a^
31	Using a working knowledge of the pathology/autopsy	1.71	1.10	32	3.97	1.16	33	0. 13
32	A professional with specialty education in forensic nursing takes charge of forensic cases	1.72	1.03	31	4.21	1.05	24	0. 10
33	Available ED forensic consultant to interface with family, police, medical examiner, etc.	1.62	1.03	33	4.38	0.91	12	0. 06
34	The forensic nurse specialist assists in searching for information in suspected cases	1.36	0.67	35	4.40	0.88	8	-0. 18^a^
35	The forensic nurse specialist consults in forensic matters for other hospital departments	1.27	0.57	37	4.19	0.97	26	-0. 22^a^
36	The forensic nurse specialist operates in the ED	1.23	0.54	39	4.31	1.00	17	-0. 21^a^
37	The forensic nurse specialist educates the staff on forensic matters	1.26	0.58	38	4.36	0.93	13	-0. 04
38	The forensic nurse specialist acts as an expert witness in court	1.31	0.66	36	4.40	0.92	9	-0. 09
39	Holding the belief that ED physicians have a strong knowledge of forensic protocol	2.46	1.27	21	4.43	0.85	6	0. 05
Total		92.07	25.48		164.82	24.86		0.11

^a^The correlation between the frequency and importance scores (*P<*0.05)

### The highest and lowest FOP and POI of FN role behaviors

The three most frequently, performed behaviors were as follows: first; informing forensic physicians about admitting a patient died on arrival without receiving care (3.66±1.56), second; assuring documentation for legal protection of the nurse/patient (3.37±1.37), and third; incorporating the collection of forensic evidence with patient care (3.14±1.36). The least frequently performed items were as follows: first; the forensic nurse specialist operating in the ED (1.23±0.54); this item refers to a registered nurse with specialized training and knowledge in forensic nursing who works within the ED of a hospital, second; the forensic nurse specialist educates the staff on forensic matters (1.26±0.58), and third; the forensic nurse specialist consults on forensic matters for other hospital departments (1.27±0.57).

The three items ranking at the top of POI of role behaviors were as follows: first; informing forensic physicians about admitting a patient died on arrival without receiving care (4.59±0.87), second; recognizing signs of physical child abuse (4.57±0.76); and third; knowing about testifying in a court of law in forensic cases (4.48±0.94). The three items perceived as the least important behaviors were as follows: first; reading dental charts and being aware of the forensic dental comparison (3.59±1.26), second; knowing the duties/responsibilities of the police investigator (3.84±1.14), and third; acting as a forensic liaison between the hospittal/victims, forensic investigators/law enforcement (3.91±1.18) (Table 2, Figs. 1 and 2).

### Correlational analysis

**Fig. 3:**
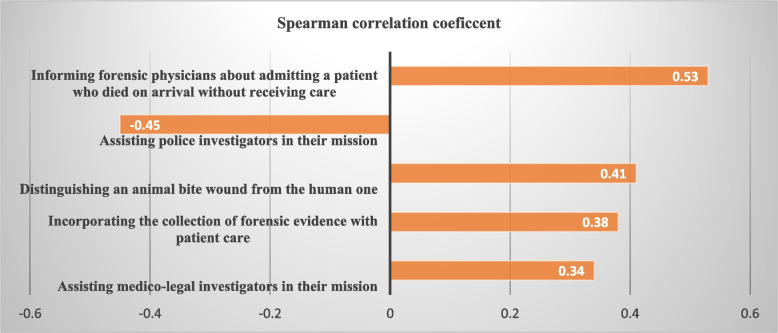
The forensic nursing role behaviors with the strongest correlation between the FOP and POI

**Fig. 4:**
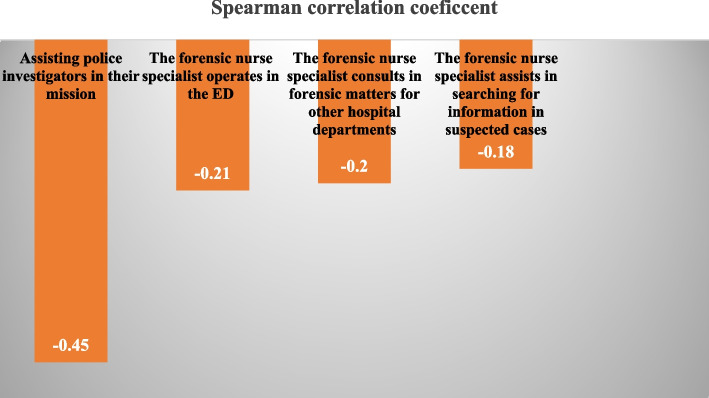
The forensic nursing role behaviors with a negative correlation between FOP and POI

The Spearman correlation test results indicated that there was a positive correlation between the frequency scores and importance scores for twenty-four items (*P<*0.05) and a negative correlation between the frequency and importance scores of four items (*P<*0.05), No significant correlation was found between FOP and POI often eleven items (*p>*0.05). There was also no significant correlation between the total FOP and POI (*p>*0.05) (Table 2). The Spearman correlation coefficient analysis revealed that the three items that had the highest correlation between FOP and POI were as follows: first; informing forensic physicians about admitting a patient who died on arrival without receiving care (*r=*0.53), second; assisting police investigators in their mission (*r=*-0.45), and third; distinguishing an animal bite wound from a human one (*r=*0.41). The more ED nurses performed these behaviors, the more they perceived them as important. The items with a negative analysis between FOP and POI were as follows: assisting police investigators in their mission (*r=*-0.45) the forensic nurse specialist operating in the ED (*r=* -0.21), and the forensic nurse specialist consulting in forensic matters for others hospitals departments (*r=* -0.020) (Table 2, Figs. 3 and 4).

There was a relationship between participants’ demographic characteristics and FOP as well as POI of FN role behavior. The mean scores of FOP positively correlated with the type of shift nurses worked and their personal experiences of legal and judicial claims. Participants who worked nightly shifts and experienced legal and judicial claims performed FN role behaviors more frequently. There was a correlation between the mean scores of POI and the female gender. Female nurses perceived FN role behaviors to be more important than male nurses (Table 3).

**Table 3:** The relationship between participants’ demographic information and FOP and POI of forensic nursing role behaviors

**Variables**	**N (%)**	**Frequency of performance**		**Perception of importance**	
		**Mean/SD**	**T- test/P**	**Mean/SD**	**T- test/P**
			**ANOVA/P**		**ANOVA/P**
**Age (year)**					
21-30	138 (50.4)	92.49±26.41	0.26	162.36±25.38	1.38
31-40	122 (44.5)	92.14±24.47	0.772	167.16±24.12	0.252
41-50	14 (5.1)	90.23±24.92		166.69±25.15	
**Gender**					
Female	192 (70.1)	91.97±25.18	0.1	166.82±22.85	2.06
Male	82 (29.9)	92.30±26.35	0.917	160.13±28.63	0.041*
**Marital status**					
Single	114 (41.6)	95.26±23.50	1.77	162.11±23.14	1.47
Married	160 (58.4)	89.79±26.73	0.078	166.62±25.94	0.143
**Education level**					
Bachelor	263 (96)	91.78±25.50	0.95	165.25±24.26	-0.07
Higher	11 (4)	98.80±26.74	0.344	152.90±38.04	0.233
**Total nursing experience (year)**					
Less than 5	90 (32.8)	94.61±24.69	0.99	165.06±23.96	1.42
10-May	110 (40.1)	93.13±26.12	0.413	164.60±24.41	0.158
15-Oct	58 (21.2)	87.07±25.14		166.33±24.45	
15-20	12 (4.4)	86.33±25.16		163.83±35.20	
20-25	3 (1.5)	87.33±34.15		148.67±41.24	
**Nursing experience in ED (month/year)**					
6-12 m	63 (23)	95.78±23.75	2.73	168.68±22.39	1.68
1-10 y	179 (65.3)	92.38±25.71	0.067	162.81±25.23	0.188
10-20 y	32 (11.7)	83.03±26.14		168.44±26.82	
**Current position**					
ED supervisor	4 (1.5)	89.25 ±33.26	0.36	166.25±20.17	0.84
ED chief nurse	5 (1.8)	103.40±14.05	0.785	169.20±22.67	0.471
ED nurse	236 (86.1)	91.81±24.98		165.53±24.37	
Triage nurse	29 (10.6)	92.66±30.38		158.03±29.44	
**Type of shift**					
In Rotation	249 (90.9)	91.22±25.33	2.82	165.05±24.97	0.53
Morning	14 (5.1)	96.43±23.71	0.039*	167.43±24.22	0.661
Evening	4 (1.5)	84.25±20.19		159.75±33.63	
Night	7 (2.6)	118.00±26.69		154.29±18.49	
**Weekly working hours**					
Less than 30	3 (1.1)	92.66±30.38	0.2	92.66±30.38	0.72
30-40	116 (42.3)	92.66±30.38	0.82	92.66±30.38	0.487
More than 40	155 (56.6)	92.66±30.38		92.66±30.38	
**Type of ED**					
General	227 (82.8)	91.54±25.65	0.92	164.95±25.25	0.78
Poisoning	17 (6.2)	96.35±29.78	0.455	167.06±19.49	0.538
Trauma	18 (6.6)	97.94±20.54		165.29±23.82	
Cardiology	13 (4.8)	85.25±22.07		156.00±26.24	
**Formal forensic nursing education**					
Yes	18 (6.6)	94.00±31.15	0.36	161.50±22.69	0.55
No	256 (93.4)	91.93±25.11	0.723	165.05±25.03	0.583
**Personal experiences of legal and judicial claims**					
Yes	16 (5.8)	105.63±21.21	2.2	164.31±25.56	0.09
No	258 (94.2)	91.23±25.53	0.028*	164.85±24.87	0.932

*P<0.05

## Discussion

The study aimed to investigate the FOP and POI of FN role behaviors of ED nurses and the distance between performed behaviors and perceived importance. Most behaviors were performed below average, with only eight items exceeding the average. Despite this, the nurses considered all role behaviors important based on the mean scores. The mean scores of the perception of importance were significantly higher than the frequency of performed role behaviors reflecting a gap between ideal and current performance. This finding aligns with the previous study by Lynch (1990), which found a gap between the frequency of role behavior performance and the value placed on behaviors by ED nurses [26].

Abdul and Brysiewicz (2009) found that ED nurses did not frequently perform FN role behaviors [27], yet they rated a high percentage of these behaviors as very important. Nurses’ practice relies on their knowledge and skills [31], and inadequate knowledge and skills in forensic issues may hinder their ability to provide quality care in forensic cases [27]. In this study, the gap between the performance of FN role behaviors and the perceived importance of ED nurses may stem from limited knowledge about FN, as the majority 93.4% of ED nurses received no education on the topic. Even participants with higher education levels did not perform FN role behaviors more frequently, indicating a lack of FN education in nursing programs. This lack of knowledge about FN has been reported in Iran [28] and other contexts [19, 32, 33]. To ensure competent forensic nursing practice, it is crucial to incorporate FN education into both undergraduate and graduate nursing curricula, as well as in-service training and courses for clinical nurses. This education should equip nurses with the necessary knowledge and skills to identify and handle cases involving violence, abuse, neglect, child and elderly abuse, sexual assault, and domestic violence. They should be able to conduct unbiased interviews, document findings discreetly, and make appropriate referrals for the victims. Additionally, nurses should be familiar with legal and ethical considerations in FN, including informed consent, confidentiality, and mandatory reporting.

The results of this study showed that the five lowest-performed role behaviors of FN were related to the forensic nurse specialist role. They included “functioning as a nurse educator to the staff in forensic matters, acting as an expert witness in court, operating primarily in the ED, acting as a consultant in forensic matters for other hospital departments, and searching for information (e.g. where child or spouse abuse is suspected). However, these role behaviors were not commonly performed, they were considered important and necessary in the EDs. In contrast to other studies where operating primarily in the ED was ranked as the fourth [26] and second [27] most frequently performed behavior, our study showed it to be the lowest-performing behavior. This difference may be due to the lack of forensic nurse specialists in Iranian clinical settings, highlighting the need for recognition of the FN specialty. A forensic nurse specialist is a dedicated nurse who provides specialized care to victims of intentional or unintentional trauma. This nurse coordinates services, conducts medical history and physical exams, takes medical-forensic photography, identifies and collects evidence, and documents victims’ conditions [2]. In Iran, FN is still in its early stages of development, but there is increasing recognition of the importance of this field. There is a need for FN programs courses, and resources to support nurses in their clinical practice which may not be readily available in EDs in Iran.

Based on the results, the first highest-performed FN role behavior was “informing forensic physicians about admitting a patient who died on arrival without receiving care”. Furthermore, this behavior ranked as the first highest-perceived importance role behavior. This item focuses on informing the medical examiner when a patient arrives at the hospital already deceased without receiving any medical treatment. The notification is crucial for forensic purposes, allowing the examiner to investigate the cause of death and gather evidence for potential legal investigations. As expected, this item showed the first strongest correlation between FOP and POI, suggesting that the more nurses engaged in this behavior, the more they perceived it as important. Lynch (1990) found similar results [26]. However, Abdul and Brysiewicz (2009) identified “the forensic nurse specialist functions as a nurse educator to the staff in forensic matters” as the first rank of FOP [27]. This may be due to adherence to existing regulations in Iranian EDs, which require referring unexplained or suspicious deaths to forensic physicians. Nurses’ fear of litigation and emphasis on their primary role may contribute to this finding. One possible explanation could be that nurses prioritize their primary role and the nature of this behavior that overlaps between the FN role and routine initial care in the ED. This may lead nurses to be more sensitive to routine care-related regulations. The current regulations appear to encourage ED nurses to adhere to a uniform set of standards, enhance their documentation and reporting practices, and increase their professional responsibility. Consequently, it is crucial to establish and implement context-specific FN regulations and protocols to ensure proper documentation, reporting, and professional responsibility.

The finding also indicated that the second highest-performed FN role behavior was “the ED nurse assures accurate documentation for investigative purposes and legal protection of both the nurse and the patient”. It indicated ED nurses frequently performed this behavior and believed it to be important. This finding can be caused by ED nurses’ concerns for protection from being accused of negligence and legal prosecution by patients and families. Nursing records are one of the most reliable documentation [34] and serve as a legal record of the care provided [35, 36]. Thus, legal issues are a critical aspect of forensic nursing practice, and ED nurses should be aware of their legal responsibilities and obligations related to reporting cases of violence or abuse.

The finding also showed the least POI role behavior was “reading dental charts and being aware of the forensic dental comparison”. The participants did not consider this behavior as important. Forensic dentistry involves identifying, documenting, recovering, and preserving all signs in the soft and hard tissues of the mouth to provide forensic evidence [37]. Lynch (1990) reported this item was included in the questionnaire as a control item to ensure the reliability of the results, and high scores were not expected. Similarly, our findings may indicate that nurses perceive this behavior beyond the scope of practice and should be performed by a forensic deontologist.

The findings showed that the item with the second strongest correlation (a negative correlation) between FOP and POI was “assisting police investigators in their mission” suggesting that the more nurses performed this behavior, the less they perceived it as important. Although ED nurses performed this behavior as the fourth frequent item, they did not perceive it as an important part of their role. This finding was confirmed by the result of the other two items regarding interaction with the judicial system including “the ED nurse has a working knowledge of the duties and responsibilities of the police investigator” and “the ED nurse is skilled in sharing information of sexual assault cases with law enforcement officer and medico-legal investigator”. These findings may suggest that nurses may lack the necessary knowledge and skills to effectively engage with the legal system. Additionally, their practice may be influenced more by their personal experiences rather than following nursing principles. Hence, it is essential to provide ED nurses with specific expertise and abilities so that they can effectively support police investigators in their work. This involves acquiring various skills, such as the ability to gather biological samples, record injuries, and safeguard any relevant physical evidence for the investigation.

According to the findings the role behavior with the third strongest correlation between FOP and POI was “distinguishing an animal bite wound from a human one” indicating that the more nurses performed this behavior, the more they perceived it as important. Similarly, Lynch 1990 and Abdul and Brysiewicz 2009 [26, 27], nurses performed this item above the average and perceived it as very important. Forensic nurses are likely to encounter a diverse range of wounds, and understanding the characteristics of gunshot wounds fired from various distances is crucial for identifying crimes. According to Johnson (2023), the presence of gunpowder on victims and suspects may be linked to self-defense and self-harm, highlighting the importance of determining the distance from which the weapon was fired [38]. It seems ED nurses did not adequately value these behaviors due to their limited knowledge, which warrants further attention.

According to the findings, the fourth strongest correlation between FOP and POI belonged to the item “incorporating a proper collection of forensic evidence with patient care for victims of trauma” reflecting that the more nurses performed this behavior, the more they perceived it as important. Participants performed this role the third most frequently even though implementation of this task requires knowledge and expertise. In contrast to this, Lynch (1990) [26] found this item eighteenth FOP rank. This inconsistency may reflect concerns about variations in non-standard practices and approaches used by ED nurses to identify and preserve evidence, which may compromise the integrity of valuable evidence [39]. Missing evidence in EDs can compromise and undermine the victim’s right to justice [26].

Furthermore, apart from the item “assisting police investigators in their mission” which showed a negative relationship between FOP and POI, three other items also exhibited a negative correlation. These items were related to the role behaviors of forensic nurse specialists, such as “operating primarily in the ED, acting as a consultant in forensic matters for other hospital departments, and searching for information”. However, participants believed that these behaviors were seldom carried out in EDs, but they still considered the presence of forensic nurse specialists and the performance of their role behaviors in EDs to be significant.

Regarding the relationship between FOP and POI of FN role behaviors with nurses’ demographic information, the two similar studies [26, 27] did not provide any information. However, this study’s findings showed that participants who worked night shifts performed FN role behaviors more frequently. This may be due to their increased exposure to trauma and violence victims during night shifts. In Iran, for example, 65% of road accidents occur at night [40]. Additionally, participants who had personal experiences with legal and judicial claims reported performing FN role behaviors more frequently, possibly due to their familiarity and sensitivity to legal and forensic issues that influence their clinical practices when dealing with forensic cases. Female participants perceived forensic nursing role behaviors as more important than male participants, which may be related to the gender differences in communication styles and interpersonal skills. Male nurses generally prefer to work in clinical settings where patients are sicker, conditions change quickly, and the workload is higher. They are more inclined to perform high-risk and stressful tasks [41] and engage in more physical activities. They are less empathetic and better equipped to deal with emergencies and can cope better with crises [42]. It is worthwhile to note, however, that these gender differences are not universal and may vary depending on individual backgrounds, experiences, and cultural norms.

## Conclusion

This highlights the need for training in FN topics, which should be integrated into nursing curricula and programs for both graduate and undergraduate students. In-service education for ED nurses is also necessary. Nurses need to be provided with the essential knowledge and abilities to recognize and manage situations related to violence, mistreatment, neglect, abuse of children and the elderly, sexual assault, and domestic violence. They should be capable of conducting impartial interviews, discreetly documenting their findings, and making suitable referrals for the individuals affected. Furthermore, considering the increasing demand for forensic nursing services in healthcare settings, specialized equipment should be taken into account for the collection and protection of important evidence in emergency departments.

Nursing leaders should provide context-based FN protocols to guide the care of trauma and violence victims, fostering collaboration between healthcare providers, law enforcement, and relevant agencies. This can lead to professional development, expertise in FN, and improved patient care and evidence collection. Researchers policymakers and administrators in the nursing field should support the growth of the forensic nursing specialty. Policymakers should make it easier for healthprofessionals in EDs to report cases of violence and refer victims for support. Furthermore, legal regulations should be in place to support nurses in their role of providing assistance to victims of violence. The findings could be relevant and suitable for Middle Eastern countries.

## Limitations

The first limitation is that the data is based on self-reports from nurses, which can be subjective and may contain recall bias or inaccuracies. Another limitation is that the survey may not cover all the behaviors or experiences of ED nurses, as it only includes the questions asked in the survey. One major concern was the uncertainty about how applicable the results would be, as the samples were collected from Iranian populations. Additionally, the findings may not apply to other settings, as the survey specifically focuses on ED nurses. The constraints within the organizational setting of emergency departments and the lack of advancement in the field of forensic nursing in Iran may have influenced the behavior of forensic nurses, potentially impacting the applicability of the findings.

## Data Availability

All data generated or analyzed during this study are included in this published article.

## Abbreviations

ED: Emergency Department
FN: Forensic Nursing
EDFNS: The Emergency Department Forensic Nursing Survey
FOP: Frequency of Performed
POI: Perception of Importance
